# Fast-MFQE: A Fast Approach for Multi-Frame Quality Enhancement on Compressed Video

**DOI:** 10.3390/s23167227

**Published:** 2023-08-17

**Authors:** Kemi Chen, Jing Chen, Huanqiang Zeng, Xueyuan Shen

**Affiliations:** 1 College of Information Science and Engineering, Huaqiao University, Xiamen 361021, China; kmchen@stu.hqu.edu.cn (K.C.);; 2 College of Engineering , Huaqiao University, Quanzhou 362021, China

**Keywords:** compressed video enhancement, lightweight models, inference time, real-time

## Abstract

For compressed images and videos, quality enhancement is essential. Though there have been remarkable achievements related to deep learning, deep learning models are too large to apply to real-time tasks. Therefore, a fast multi-frame quality enhancement method for compressed video, named Fast-MFQE, is proposed to meet the requirement of video-quality enhancement for real-time applications. There are three main modules in this method. One is the image pre-processing building module (IPPB), which is used to reduce redundant information of input images. The second one is the spatio-temporal fusion attention (STFA) module. It is introduced to effectively merge temporal and spatial information of input video frames. The third one is the feature reconstruction network (FRN), which is developed to effectively reconstruct and enhance the spatio-temporal information. Experimental results demonstrate that the proposed method outperforms state-of-the-art methods in terms of lightweight parameters, inference speed, and quality enhancement performance. Even at a resolution of 1080p, the Fast-MFQE achieves a remarkable inference speed of over 25 frames per second, while providing a PSNR increase of 19.6% on average when QP = 37.

## 1. Introduction

**Figure 1:**
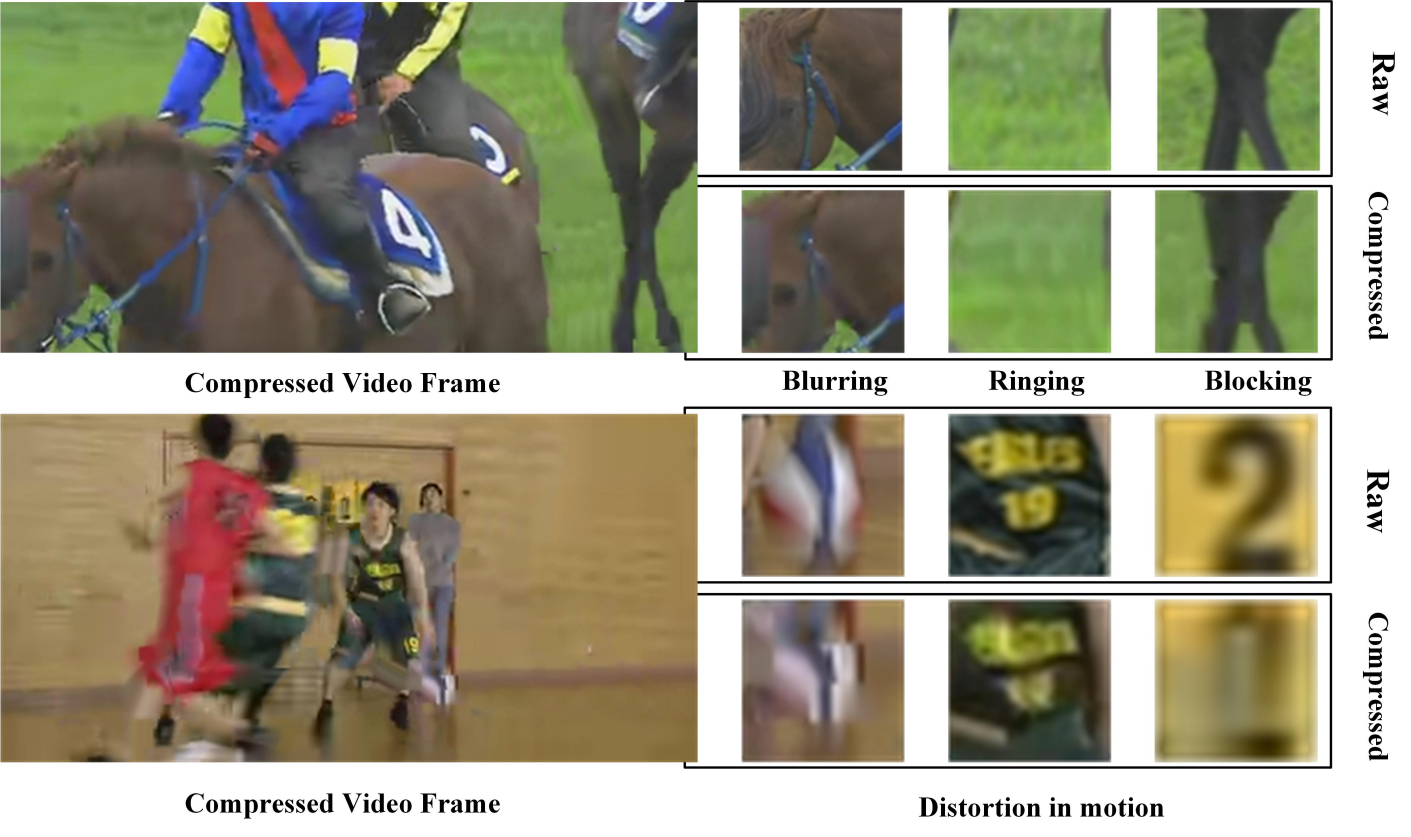
Artifacts of compressed video at 240p and QP = 37.

Nowadays, there is a surplus of ultra-high-definition (UHD) videos accessible for online viewing, imposing substantial strain on communication bandwidth. To transmit videos within the constraint of limited network bandwidth, video compression is vital for reducing the bit rate. However, highly efficient video coding standards, such as H.264/AVC [1] and H.265/HEVC [2], introduce artifacts when using de-correlation and predictive coding techniques, degrading the quality of the video to some extent [3]. As illustrated in Figure 1, after being transmitted in low bandwidth, the reconstructed video is of low quality. The artifacts (i.e., blurring, ringing, blocking and distortion in motion, etc.) are obvious. When these videos are used for subsequent visual tasks, such as object recognition, object detection, and object tracking, etc., the low quality affects the performance dramatically [4,5]. Therefore, quality enhancement for compressed video is crucial for video applications and has emerged as a crucial area of research.

For image or single-frame quality enhancement, traditional methods [6,7,8,9,10,11] aimed to enhance the quality of compressed JPEG images by optimizing the transform coefficients of a specific compression standard. Specifically, refs. [8,9] proposed Shaped-Adaptive DCT (SADCT) and Regression Tree Fields (RTF) to reduce JPEG image blocking artifacts, respectively. Nevertheless, it is challenging to apply these methods to other compression tasks due to the limited generalization ability. With the advance of the deep learning method, an expanding range of methods have embraced convolutional neural network (CNN) approaches [12,13,14,15,16] to improve the compressed image quality. In [12], a four-layer AR-CNN was first introduced to deal with various artifacts in JPEG images. Based on this, Zhang et al. [13] proposed a deep DnCNN for multi-image restoration. Then, based on residual non-local attention, a method named RNAN [17] was proposed to eliminate the image noise. Subsequent methodologies included the use of recursive units and gate units to remove JPEG artifacts [18], as well as the implementation of a dual-stream multi-path recursive residual network [19]. Later on, Lin et al. [20] proposed a multiscale image fusion approach to remove JPEG artifacts effectively, and achieved exceptional objective quality. But these methods cannot be extended to compressed video directly, since they treat frames independently and thus fail to exploit temporal information.

To enhance the quality of the compressed video, a 10-layer CNN automatic decoder (DCAD) [21] was the first work to mitigate distortion in compressed videos. In [22], two sub-networks of DS-CNN were introduced to address both intra-frame and inter-frame artifacts. Their main purpose was to enhance the target frame by leveraging the spatial correlation between video frames. There are many multi-frame compressed video enhancement methods [23,24,25,26]. Yang et al. [23] introduced a multi-frame quality enhancement network, named MFQE1.0, which leveraged adjacent high-quality frames to enhance the target frame. MFQE2.0 [24] was an improved version. And then, QG-ConvLSTM [25], a method utilizing bidirectional recurrent convolution, to capture the extensive temporal information. Based on the deformable convolution (DCN), Deng et al. [26] introduced patio-temporal deformable convolution (STDF) to extract temporal information from multiple frames by expanding the input frames to 7 or even 9 frames effectively. These methods aimed to enhance the target frame by leveraging the temporal relationships among multiple video frames primarily. Generally, multi-frame compressed video enhancement tended to achieve better results compared to single-frame enhancement due to its utilization of richer temporal and spatial information. However, these methods for compressed video enhancement faced the following challenges:

(1) The parameters of networks are excessively large, which challenges the efficiency of the training and real-time tasks.

(2) Existing methods tend to prioritize enhanced results at the expense of inference speed.

Therefore, it is necessary to explore lightweight and high-performance models for compressed video quality enhancement.

The term “lightweight model” refers to compressing the model size to maximize computational speed while preserving the accuracy. Researchers have been paying increasing attention to developing lightweight models in the field of image classification to enable deployment on mobile devices [14,15,16,27,28]. Among the pioneer endeavors in developing lightweight models, SqueezeNet [29] emerged, replacing 3 × 3 convolutions with 1 × 1 convolutions, resulting in a parameter reduction of approximately one-fiftieth compared to AlexNet [30]. Subsequently, Xception [31] further reduced the parameters by decoupling the Inception structure [32]. The ResNeXt [33] introduced group convolutions and reduced the parameters by integrating the residual network and Inception structure [32] effectively. In 2017, Google introduced MobileNet [34], which pioneered the concept of depthwise separable convolution (DSC) to effectively reduce the parameters in neural networks. Subsequently, MobileNet V2 [35] surpassed the previous performance benchmarks by implementing inverted residual structures and linear bottleneck layers. After that, ShuffleNet [36,37] reduced the model parameters by successfully employing group convolutions and enabling inter-channel interaction via channel shuffling operations. More recently, Huang et al. introduced CondenseNet [38], a novel approach that combines model pruning and group convolutions to effectively reduce the number of model parameters. However, these methods were not used for video quality enhancement (VQE).

To enhance the quality of compressed video and achieve superior inference performance, an end-to-end CNN-based method for VQE task, named Fast-MFQE, is proposed. The main contributions of the method are as follows:

(1) A novel IPPB module is designed to reduce the multi-frame information redundancy and fasten the inference speed;

(2) STFA and FRN modules are proposed to effectively extract the temporal features and the multi-frame correlation.

**Figure 2:**
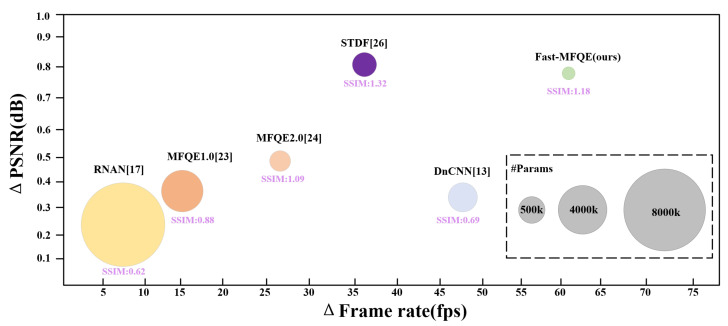
Inference speed and performance comparison of VQE methods.

More intuitively, the parameters and the performance of diverse VQE methods are shown in Figure 2. It can be seen that compared to state-of-the-art VQE methods, the proposed Fast-MFQE method demonstrates smaller parameters and superior inference performance, as well as improving the quality of the compressed video, such as ΔPSNR and ΔSSIM $\times \;10^{-2}$. The structure of the remaining sections in this paper is as follows: Section 2 provides a detailed exposition of the proposed method. Section 3 presents the experimental results and provides an analysis of the superior performance of the proposed method. Section 4 concludes the paper.

## 2. The Proposed Fast-MFQE

**Figure 3:**
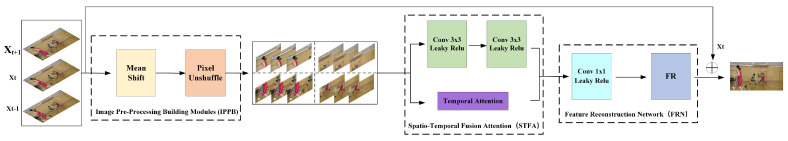
Architecture of the proposed Fast-MFQE.

The architecture of the proposed Fast-MFQE is shown in Figure 3, where Depthwise Separable Convolution (DSC) [34] is employed in place of traditional convolution to decrease the computational complexity and enhance the inference speed of the neural network. The primary objective of Fast-MFQE is to generate an enhanced video frame ${\hat{O}}_{t}$ that closely resembles the Ground-truth frame in the pixel domain. The Ground truth refers to the original uncompressed video frame. To leverage temporal information from adjacent frames, Fast-MFQE takes the target frame $V_{t}$ and its neighboring frame ${\left\{V_{t\pm n}\right\}}_{n=1}^{N}$ as the input of the network. There are three main models in Fast-MFQE; each will be illustrated in the following subsections.

### 2.1. Image Pre-Processing Building Modules (IPPB)

There are prevalent approaches in compressed video enhancement that utilize multiple video frames as input to effectively incorporate temporal information from diverse frames. However, these networks encounter challenges in achieving rapid inference when processing high-resolution video frames due to the substantial increase in computational complexity. Consequently, pre-processing of the input frames becomes imperative to facilitate fast inference in high-resolution video.

To leverage the information from adjacent frame ${\left\{V_{t\pm n}\right\}}_{n=1}^{N}$ to enhance the target frame $V_{t}$, Fast-MFQE utilizes both ${\left\{V_{t\pm n}\right\}}_{n=1}^{N}$ and $V_{t}$ as inputs to the network. To decrease the data volume of the input frames and enhance the model’s inference speed, Fast-MFQE introduces the Image Pre-Processing Building Modules (IPPB) inspired by [34,35]. As depicted in Figure 3, IPPB consists of two primary components: Mean Shift and Pixel Unshuffle.

#### 2.1.1. Mean Shift

In general, video frames exhibit substantial spatial redundancy, and mitigating this redundancy can reduce input data effectively. In a groundbreaking work, Zhang et al. [39] first introduced the Mean Shift operation to image super-resolution tasks and presented the RCAN network, which achieved remarkable results. The Mean Shift operation serves to normalize data by emphasizing individual differences by subtracting the statistical mean value from each image sample. Drawing inspiration from [37,39], Fast-MFQE employs the Mean Shift operation to diminish redundant information in images, enhancing model training speed and consequently reducing the inference time.

Let Fast-MFQE take the adjacent frame ${\left\{V_{t\pm n}\right\}}_{n=1}^{N}$ and the target frame $V_{t}$ as the network input (n = 3), with Mean Shift operation denoted as $MS\left(\cdot \right)$. Then, the feature after Mean Shift operation $F_{MS}$ can be expressed as:

(1) $$F_{MS}=MS\left({\left\{V_{t\pm n}\right\}}_{n=1}^{N},V_{t}\right)$$

#### 2.1.2. Pixel Unshuffle

While reducing redundancy in individual video frames through mean shift operations is effective, downsampling the input data is necessary to further alleviate the computational burden on the network.

Inspired by the FFDnet network [40], Fast-MFQE employs a reversible downsample (R-Downsample) operation to divide the input frames into four sub-frames, aiming to reduce the input data volume within the network. This operation decreases the model’s computational cost and inference time while effectively preserving more detailed information. Consequently, it facilitates improved model performance and enhances the generalization ability. It is worth noting that the inverse operation of R-Downsample is denoted as R-Upsample.

Let the four sub-frames generated by the R-Downsample operation be denoted as $I_{l}$ (*l* = 1, 2, 3, 4), with the R-Downsample denoted as $RD\left(\cdot \right)$. Then, the $I_{l}$ can be expressed as:

(2) $$I_{l}=RD\left(F_{MS}\right)$$

### 2.2. Spatio-Temporal Attention Fusion (STAF)

**Figure 4:**
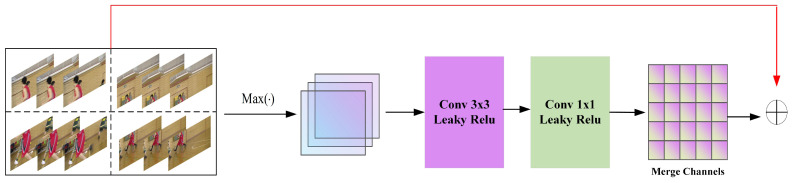
Architecture of Spatio-Temporal Attention Fusion (STAF).

Spatio-temporal information of video frames is essential for quality enhancement. To enhance the fusion of image information from different temporal and spatial contexts, Fast-MFQE introduces the Spatio-Temporal Attention Fusion (STAF) module. This module consists of two 3 × 3 convolutions that extract spatial information from the video frames. Subsequently, temporal attention extraction is performed to capture the temporal characteristics of the frames, as described in Figure 4. Then, the spatial and temporal information is fused by concatenating in the manner of channel dimension. Finally, the concatenated information undergoes fusion through a 1 × 1 convolutional layer. This process ensures the effectiveness of the spatial and temporal integration for compressed video enhancement. Let the spatial information extracted by the two 3 × 3 convolutions be denoted as $F_{S}$ and the temporal information extracted by temporal attention be denoted as $F_{T}$. The fused spatio-temporal feature is denoted as $F_{ST}$ by the 1 × 1 convolution. These features are formulated as follows:

(3) $$\begin{array}{c} F_{S}=Conv3\left(Conv3\left(\left[I_{1},I_{2},I_{3},I_{4}\right]\right)\right) \end{array}$$

(4) $$\begin{array}{c} F_{T}=TA\left(\left[I_{1},I_{2},I_{3},I_{4}\right]\right) \end{array}$$

(5) $$\begin{array}{c} F_{ST}=Conv1\left(\left[F_{S},F_{T}\right]\right) \end{array}$$

where $Conv3\left(\cdot \right)$ and $Conv1\left(\cdot \right)$ are the mapping functions of 3 × 3 convolution and 1 × 1 convolution, respectively. [·,·] represents the concatenation operation, and TA(·) is the mapping function of temporal attention.

### 2.3. Feature Reconstruction Network (FRN)

**Figure 5:**
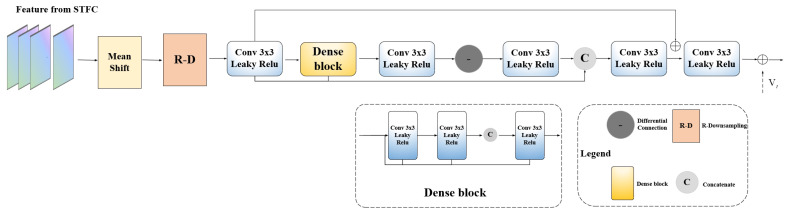
Architecture of Feature Reconstruction Network (FRN).

To achieve precise reconstruction of video frames, the Feature Reconstruction Network (FRN) is introduced in Fast-MFQE. As shown in Figure 5, the FRN consists of dense residual blocks, primarily difference learning and residual learning. Difference learning is dedicated to capturing high-frequency information in video frames by calculating element-wise differences in feature maps. And residual learning aims to learn diverse feature information by calculating element-wise additions of feature maps. By incorporating both difference and residual learning, relevant features are captured and integrated effectively, enabling the generation of refined video frames. Let $R_{t}$ denote the reconstructed information generated by the FRN while $V_{t}$ represents the target frame; the reconstructed frames, denoted as $R_{t}^{HQ}$, can be expressed as follows:

(6) $$R_{t}^{HQ}=R_{t}+V_{t}$$

### 2.4. Loss Function

To encourage the enhanced frame $R_{t}^{HQ}$ to be as close as possible to the original uncompressed frame $V_{raw}$ in the pixel domain, Fast-MFQE adopts the mean square error between the enhanced frame $R_{t}^{HQ}$ and the original uncompressed frame $V_{raw}$ as the loss function of the model, which is formulated as follows:

(7) $$L\left(\theta \right)=\frac{1}{HWC}{\left|\left|R_{t}^{HQ}-V_{raw}\right|\right|}_{2}^{2}$$

where *H*, *W*, and *C* represent the height, width, and number of channels of the image under evaluation, respectively. θ can be learned through the gradient descent algorithm [41] to solve Equation (7). Thus, Fast-MFQE can be trained effectively to enhance the quality of compressed videos.

## 3. Experiments

In this section, the effectiveness of the proposed Fast-MFQE method is demonstrated by extensive experiments. The experimental settings are introduced in Section 3.1, and the performance comparisons of the Fast-MFQE method with state-of-the-art methods for JCT-VC testing sequences [2] are illustrated in Section 3.2.

### 3.1. Settings

#### 3.1.1. Datasets

The Fast-MFQE model is trained using the dataset introduced in MFQE2.0 [24]. The dataset [24] is divided into two parts. Firstly, 18 sequences from the Joint Collaborative Team on Video Coding (JCT-VC) [2] are commonly utilized as a test set. Secondly, the remaining 142 random sequences are split into non-overlapping training (106 sequences) and validation (36 sequences) sets. All 160 sequences are compressed using HM16.5 [1] in Low-Delay configuration, which is an encoding platform for H.265, with QP set to 27, 32, and 37. The results demonstrate the excellent generalization ability of the proposed method, making it applicable to different QP values.

#### 3.1.2. Quality Enhancement Assessment Metrics

Extensive research [13,42,43,44] has been conducted to develop efficient and accurate methods for assessing the quality of images and video frames. The quality enhancement evaluation metric is used to measure the quality of distorted images or video frames by comparing the corresponding ground truth quantitatively using full-reference evaluation metrics. In this experiment, two widely used evaluation metrics, namely PSNR and SSIM [42], are employed.

#### 3.1.3. Parameter Settings

The basic settings and hyperparameters of the experiments are presented here. The Fast-MFQE model is trained using the PyTorch framework. Specifically, the Fast-MFQE takes three frames (n = 3) as input to the network. The iteration number is $3\times 10^{5}$ and the mini-batch size is 32. The cropped size is reduced to 128 × 128. The learning rate is set to $1\times 10^{-4}$ and halved every $1\times 10^{5}$ iterations. Note that the above hyperparameters are tuned over the training set. Finally, the parameters of the Fast-MFQE network are updated utilizing the Adam algorithm [41] until the network converges.

### 3.2. Performance Comparison

#### 3.2.1. Quantitative Comparison

In this section, the performance of the Fast-MFQE model is evaluated using Peak Signal-to-Noise Ratio (PSNR) and Structure Similarity Index Measure (SSIM) quantitatively, which are used objective quality evaluation metrics widely. The Fast-MFQE model is compared with AR-CNN [12], DnCNN [13], RNAN [17], MFQE1.0 [23], and MFQE2.0 [24]. Among these methods, AR-CNN [12], DnCNN [13], and RNAN [17] are methods for enhancing the quality of compressed images, MFQE1.0 [23] is the first method used for multi-frame compressed video enhancement, and MFQE2.0 [24] is the most advanced method for enhancing the quality of compressed videos. To ensure a fair comparison, all of these methods are trained and tested on the same dataset.

Table 1 reports the ΔPSNR and ΔSSIM results, which are calculated between enhanced and compressed frames averaged over each test sequence. Note that ΔPSNR > 0 and ΔSSIM > 0 indicate improvement in objective quality for the compressed video. Specifically, compared to the most advanced compressed video enhancement method, MFQE2.0 [24], Fast-MFQE achieves an average increase of 19.6% in PSNR and an average increase of 8.2% in SSIM at QP = 37, and an average increase of 12.2% in PSNR and an average increase of 14.2% in SSIM at QP = 27. Although the enhancement effect of Fast-MFQE is close to that of MFQE2.0 [24], the inference speed of Fast-MFQE is faster. Overall, The Fast-MFQE outperforms all compared methods in terms of objective quality enhancement.

#### 3.2.2. Subjective Comparison

**Figure 6:**
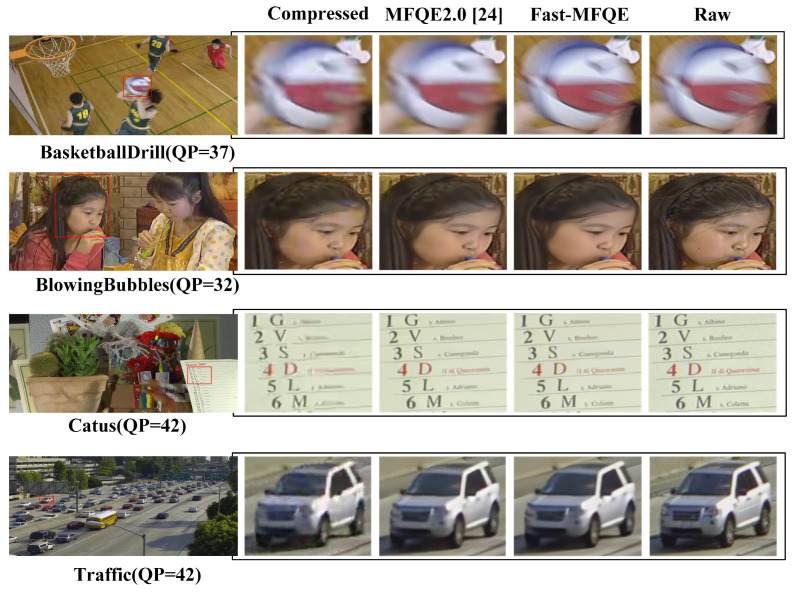
Subjective quality comparison on *BasketballDrill* at QP = 37, *BlowingBubbles* at QP = 32, *Catus* and *Traffic* at QP = 42. It can be seen that the Fast-MFQE approach achieves a clearer effect than the most advanced compressed video enhancement method MFQE2.0 [24].

In this section, the subjective evaluation of the Fast-MFQE is mainly focused on the following. As shown in Figure 6, the video frames are enhanced as follows: *BasketballDrill* at QP = 37, *BlowingBubbles* at QP = 32, *Catus* and *Traffic* at QP = 42. It can be observed that the proposed Fast-MFQE method has sharper edges and more vivid details than other methods. For example, the basketball in *BasketballDrill*, the face in *BlowingBubbles*, the words in *Catus*, and the car in *Traffic* can be restored with fine textures in Fast-MFQE, which is similar to MFQE2.0 [24].

#### 3.2.3. Comparison of Inference Performance

In this section, the inference capability and the degree of lightweight of the Fast-MFQE model are quantitatively evaluated based on the frame rate and amount of parameters (Param). The Fast-MFQE model is compared with DnCNN [7], RNAN [17], MFQE1.0 [23], MFQE2.0 [24], and STDF [26]. It should be noted that STDF [26] is currently the most lightweight model used for compressed video enhancement. All models are tested on the following configurations: 11th Gen Intel(R) Core(TM) i7-11800H @ 2.30GHz 2.30 GHz, Nvidia GeForce GTX 1080Ti GPU, and Ubuntu 20.04 for the sake of fairness.

Table 2 reports the number of parameters (Param) and the frame rate of different models. The Fast-MFQE maintains a speed of over 25 frames per second for all resolution videos. Notably, even when processing 1080p resolution videos, the Fast-MFQE achieves a speed close to 25 frames per second, ensuring smooth and non-stuttering video processing. Additionally, the Fast-MFQE reduces the parameters by 33.4% compared to the current lightest model, STDF [26]. Overall, the Fast-MFQE outperforms the compared methods in terms of model inference speed and parameters.

#### 3.2.4. Ablation Studies

As shown in Table 3, the ablation test was performed at QP = 37. Although Model2 and Model3 have significantly improved the Inference speed, the enhancement effects, such as ΔPSNR and ΔSSIM, are declining sharply. Therefore, in order to better balance the enhancement effect and Inference speed, we choose Model1; that is, the three modules of IPPB, STFA, and FRN are all necessary.

#### 3.2.5. Perceptual Quality Comparison

In this section, the performance of the Fast-MFQE is evaluated quantitatively using the learned perceptual image patch similarity (LPIPS) [45] and perceptual index (PI) [46], which are widely used perceptual quality assessment metrics. Table 4 reports the ΔLPIPS and ΔPI results, which are calculated between enhanced and compressed frames averaged over each test sequence. Note that ΔLPIPS <0 and ΔPI <0 indicate improvement in perceptual quality. As shown in this table, the Fast-MFQE is significantly superior to all the compared methods in terms of perceptual quality enhancement.

#### 3.2.6. Subjective Quality and Inference Speed at Different Resolutions

**Figure 7:**
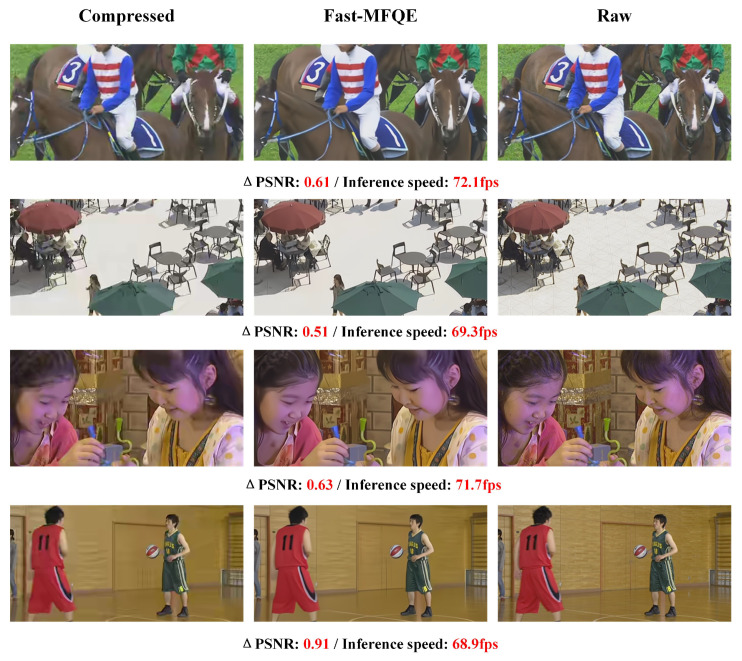
Subjective quality and inference speed at 240p.

**Figure 8:**
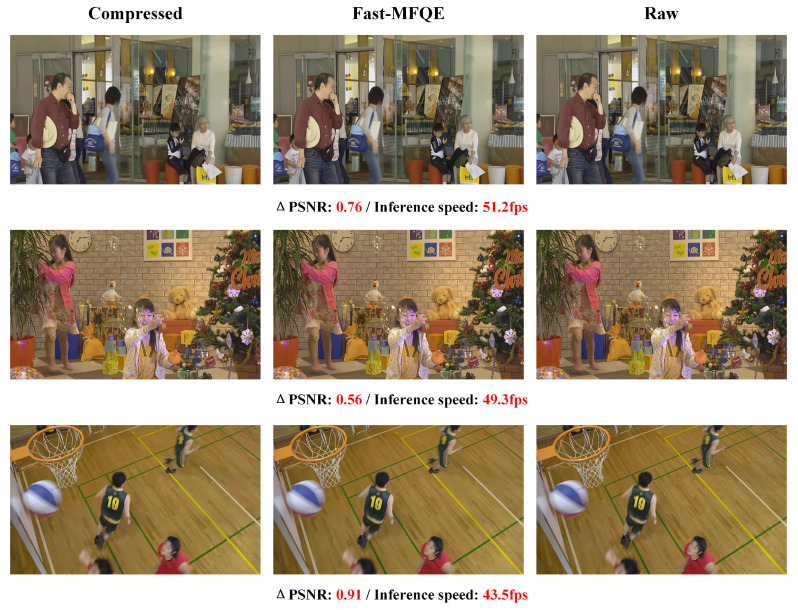
Subjective quality and inference speed at 480p.

**Figure 9:**
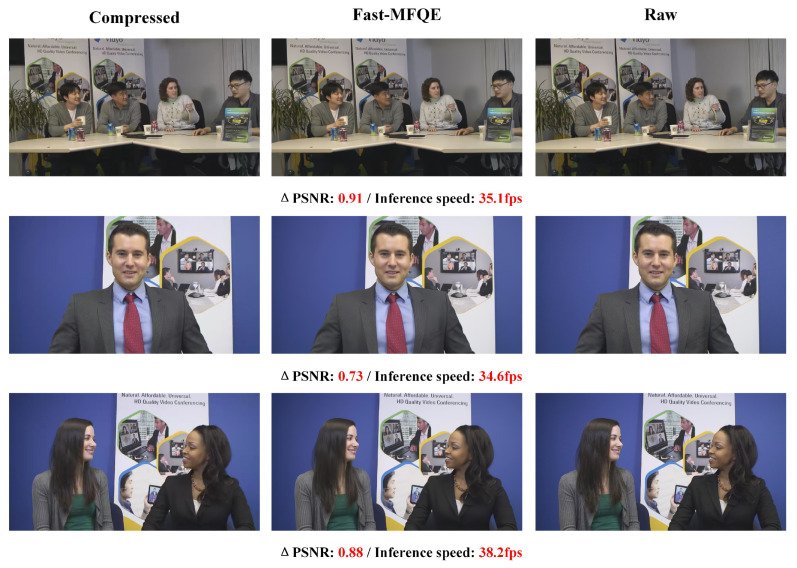
Subjective quality and inference speed at 720p.

**Figure 10:**
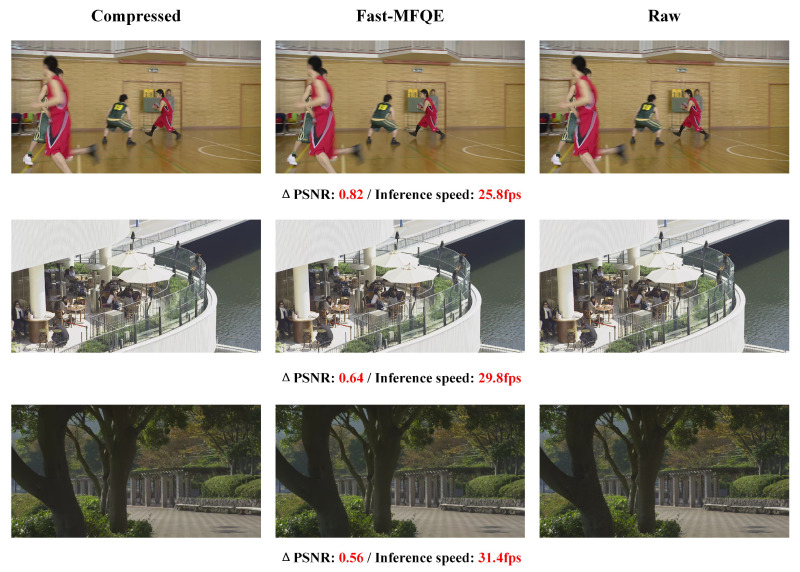
Subjective quality and inference speed at 1080p.

**Figure 11:**
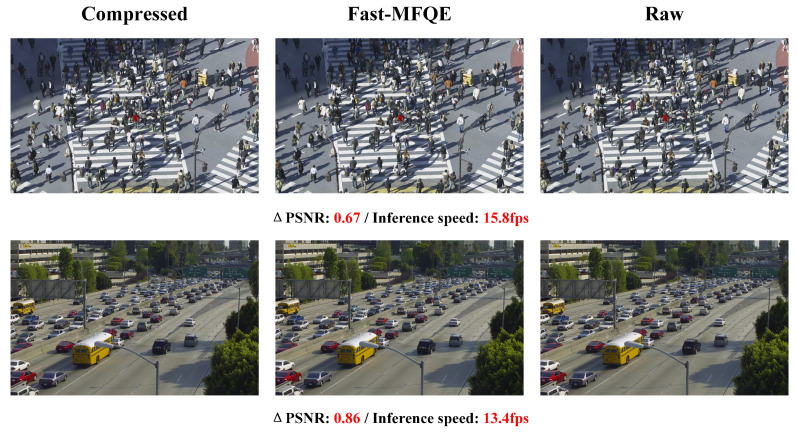
Subjective quality and inference speed at 1600p.

This section focuses on the performance of the proposed Fast-MFQE regarding inference and enhancement across videos with different resolutions. Note that these videos are all tested at QP = 37. As shown in Figure 7, Figure 8, Figure 9, Figure 10 and Figure 11, we test the inference speed and performance of the model at different resolutions and QP = 37. The results demonstrate that the Fast-MFQE is capable of performing fast inference with high quality across different resolutions.

## 4. Conclusions

This paper presents a fast multi-frame quality enhancement approach, named Fast-MFQE, which facilitates efficient model inference. The Fast-MFQE is the first lightweight model in the field of compressed video enhancement. Extensive experiments demonstrate that the Fast-MFQE outperforms previous methods in terms of its lightweight parameters, fast inference speed, and quality enhancement performance on benchmark datasets. Its remarkable attributes make it an ideal solution for real-time applications such as video streaming, video conferencing, and video surveillance, unlocking a range of possibilities in these domains.

## Figures and Tables

**Table 1:** Overall comparison for ΔPSRN(dB) and ΔSSIM ($\times 10^{-2}$) over test sequences at three QPs.

QP	Video Sequence		AR-CNN [12]		DnCNN [13]		RNAN [17]		MFQE1.0 [23]		MFQE2.0 [24]		Fast-MFQE	
			ΔPSNR↑	ΔSSIM↑	ΔPSNR↑	ΔSSIM↑	ΔPSNR↑	ΔSSIM↑	ΔPSNR↑	ΔSSIM↑	ΔPSNR↑	ΔSSIM↑	ΔPSNR↑	ΔSSIM↑
37	A	Traffic	**0.27**	**0.50**	**0.35**	**0.64**	**0.40**	**0.86**	**0.50**	**0.90**	**0.59**	**1.02**	**0.61**	**1.23**
		PeopleOnStreet	**0.37**	**0.76**	**0.54**	**0.94**	**0.74**	**1.30**	**0.80**	**1.37**	**0.92**	**1.57**	**0.97**	**1.67**
	B	Kimono	**0.20**	**0.59**	**0.27**	**0.73**	**0.33**	**0.98**	**0.50**	**1.13**	**0.55**	**1.18**	**0.66**	**1.23**
		ParkScene	**0.14**	**0.44**	**0.17**	**0.52**	**0.20**	**0.77**	**0.39**	**1.03**	**0.46**	**1.23**	**0.53**	**1.33**
		Cactus	**0.20**	**0.41**	**0.28**	**0.53**	**0.35**	**0.76**	**0.44**	**0.88**	**0.50**	**1.00**	**0.64**	**1.16**
		BQTerrace	**0.23**	**0.43**	**0.33**	**0.53**	**0.42**	**0.84**	**0.27**	**0.48**	**0.40**	**0.67**	**0.52**	**0.86**
		BasketballDrive	**0.23**	**0.51**	**0.33**	**0.63**	**0.43**	**0.92**	**0.41**	**0.80**	**0.47**	**0.83**	**0.74**	**0.91**
	C	RaceHourse	**0.23**	**0.49**	**0.31**	**0.70**	**0.39**	**0.99**	**0.34**	**0.55**	**0.39**	**0.80**	**0.53**	**0.93**
		BQMall	**0.28**	**0.69**	**0.38**	**0.87**	**0.45**	**1.15**	**0.51**	**1.03**	**0.62**	**1.20**	**0.72**	**1.23**
		PartyScene	**0.14**	**0.52**	**0.22**	**0.69**	**0.30**	**0.98**	**0.22**	**0.73**	**0.36**	**1.18**	**0.44**	**1.31**
		BasketballDrill	**0.23**	**0.48**	**0.42**	**0.89**	**0.50**	**1.07**	**0.48**	**0.90**	**0.58**	**1.20**	**0.63**	**1.26**
	D	RaceHorses	**0.26**	**0.59**	**0.34**	**0.80**	**0.42**	**1.02**	**0.51**	**1.13**	**0.59**	**1.43**	**0.68**	**1.47**
		BQSquare	**0.21**	**0.30**	**0.30**	**0.46**	**0.32**	**0.63**	**-0.01**	**0.15**	**0.34**	**0.65**	**0.47**	**0.68**
		BlowingBubles	**0.16**	**0.46**	**0.25**	**0.76**	**0.31**	**1.08**	**0.39**	**1.20**	**0.53**	**1.70**	**0.61**	**1.89**
		BasketballPass	**0.26**	**0.63**	**0.38**	**0.83**	**0.46**	**1.08**	**0.63**	**1.38**	**0.73**	**1.55**	**0.88**	**1.67**
	E	FourPeople	**0.40**	**0.56**	**0.54**	**0.73**	**0.70**	**0.97**	**0.66**	**0.85**	**0.73**	**0.95**	**0.87**	**0.97**
		Johnny	**0.24**	**0.21**	**0.47**	**0.54**	**0.56**	**0.88**	**0.55**	**0.55**	**0.60**	**0.68**	**0.71**	**0.73**
		KristenAndSara	**0.41**	**0.47**	**0.59**	**0.62**	**0.63**	**0.80**	**0.66**	**0.75**	**0.75**	**0.85**	**0.86**	**0.88**
		Average	**0.25**	**0.50**	**0.36**	**0.69**	**0.41**	**0.62**	**0.46**	**0.88**	**0.56**	**1.09**	**0.67**	**1.18**
32		Average	**0.19**	**0.17**	**0.33**	**0.41**	**/**	**/**	**0.43**	**0.58**	**0.52**	**0.68**	**0.63**	**0.69**
27		Average	**0.16**	**0.09**	**0.33**	**0.26**	**/**	**/**	**0.40**	**0.34**	**0.49**	**0.42**	**0.55**	**0.48**

**Table 2:** Inference speed at different resolutions and number of parameters(param).

		Inference Speed(f/s)					Param(k)
	**Res.**	**120p**	**240p**	**480p**	**720p**	**1080p**	
**Method**							
DnCNN [13]		191.8	54.7	14.1	6.1	2.6	556
RNAN [17]		5.6	3.2	1.4	0.6	0.08	8957
MFQE1.0 [23]		34.3	12.6	3.8	1.6	0.7	1788
MFQE2.0 [24]		56.5	25.3	8.4	3.7	1.6	255
STDF [26]		13.27	36.4	9.1	3.8	1.6	365
**Fast-MFQE**		**162.1**	**60.3**	**43.1**	**32.3**	**25.7**	**243**

**Table 3:** Ablation studies at QP = 37.

Model	Model1	Model2	Model3
**IPPB**	Yse	No	No
**STFA**	Yes	Yes	No
**FRN**	Yes	Yes	Yes
Δ**PSNR/**Δ**SSIM (**$\times 10^{-2}$)	0.68/1.19	0.42/0.89	0.21/0.45
**Inference speed(f/s)**	32.1	45.3	73.2

**Table 4:** Perceptual Quality Comparison.

QP	Video Sequence		AR-CNN [12]		DnCNN [13]		RNAN [17]		MFQE1.0 [23]		MFQE2.0 [24]		Fast-MFQE	
			ΔLPIPS↓	ΔPI↓	ΔLPIPS↓	ΔPI↓	ΔLPIPS↓	ΔPI↓	ΔLPIPS↓	ΔPI↓	ΔLPIPS↓	ΔPI↓	ΔLPIPS↓	ΔPI↓
**37**	**A**	**Traffic**	0.028	0.720	0.027	0.653	0.026	0.644	0.027	0.593	0.023	0.572	0.018	0.569
		**PeopleOnStreet**	0.029	0.726	0.028	0.631	0.029	0.643	0.026	0.631	0.019	0.539	0.017	0.520
	**B**	**Kimono**	0.030	0.733	0.031	0.664	0.032	0.657	0.029	0.622	0.020	0.617	0.018	0.639
		**ParkScene**	0.028	0.728	0.032	0.635	0.031	0.624	0.028	0.572	0.024	0.691	0.021	0.701
		**Cactus**	0.031	0.699	0.026	0.586	0.025	0.590	0.027	0.630	0.030	0.616	0.027	0.593
		**BQTerrace**	0.029	0.746	0.027	0.614	0.028	0.573	0.027	0.621	0.026	0.593	0.028	0.582
		**BasketballDrive**	0.030	0.732	0.031	0.720	0.029	0.680	0.030	0.675	0.025	0.623	0.021	0.641
	**C**	**RaceHourses**	0.027	0.751	0.026	0.709	0.025	0.716	0.031	0.695	0.018	0.641	0.019	0.611
		**BQMall**	0.032	0.638	0.031	0.682	0.032	0.644	0.024	0.632	0.017	0.671	0.028	0.576
		**PartScene**	0.027	0.699	0.028	0.627	0.027	0.609	0.025	0.594	0.026	0.617	0.030	0.548
		**BasketballDrill**	0.032	0.721	0.031	0.662	0.032	0.614	0.030	0.601	0.027	0.614	0.021	0.509
	**D**	**RaceHourse**	0.030	0.758	0.029	0.631	0.028	0.629	0.027	0.622	0.030	0.601	0.019	0.627
		**BQSquare**	0.029	0.771	0.029	0.691	0.027	0.678	0.025	0.631	0.029	0.597	0.020	0.561
		**BlowingBubles**	0.032	0.712	0.031	0.786	0.032	0.645	0.031	0.591	0.022	0.596	0.023	0.558
		**BasketballPass**	0.031	0.733	0.027	0.673	0.028	0.593	0.032	0.573	0.023	0.610	0.026	0.606
	**E**	**FourPeople**	0.026	0.726	0.028	0.765	0.027	0.712	0.026	0.670	0.022	0.632	0.021	0.617
		**Johnny**	0.028	0.761	0.027	0.668	0.027	0.623	0.028	0.640	0.024	0.621	0.023	0.618
		**KristenAndSara**	0.031	0.715	0.030	0.719	0.031	0.639	0.030	0.627	0.026	0.615	0.024	0.597
		**Average**	**0.029**	**0.726**	**0.028**	**0.673**	**0.028**	**0.639**	**0.027**	**0.623**	**0.023**	**0.614**	**0.022**	**0.592**
**32**		**Average**	**0.028**	**0.564**	**0.026**	**0.533**	**0.023**	**0.515**	**0.023**	**0.501**	**0.020**	**0.495**	**0.018**	**0.493**
**27**		**Average**	**0.026**	**0.377**	**0.024**	**0.345**	**0.022**	**0.386**	**0.021**	**0.374**	**0.019**	**0.326**	**0.017**	**0.314**

## Data Availability

The data used to support the findings of this study are available from the corresponding author upon request.

## Abbreviations

The following abbreviations are used in this manuscript:

IPPB	Image pre-processing building module.
STFA	Spatio-temporal fusion attention.
FRN	Feature reconstruction network.
UHD	Ultra-high-definition.
QoE	Quality of experience.
CNN	Convolutional neural network.
PSNR	Peak Signal-to-Noise Ratio.
SSIM	Structural Similarity Index Measure.
DSC	Depthwise Separable Convolution.
